# Enhanced Recovery of Natural Antioxidants from Grape Waste Using Natural Eutectic Solvents-Based Microwave-Assisted Extraction

**DOI:** 10.3390/molecules28031153

**Published:** 2023-01-24

**Authors:** Raquel Cañadas, Blanca Sáenz de Miera, Paloma Méndez, Emilio J. González, María González-Miquel

**Affiliations:** Department of Chemical and Environmental Engineering, Higher Technical School of Industrial Engineering, Universidad Politécnica de Madrid. C/ José Gutiérrez Abascal 2, 28006 Madrid, Spain

**Keywords:** natural eutectic solvents, green extraction processes, solid–liquid extraction, microwave-assisted extraction, grape waste, phenolic compounds, natural antioxidants, flavonoids

## Abstract

The evaluation of sustainable solvents as alternatives to more harmful conventional solvents combined with intensification techniques to recover phenolic compounds from agri-food waste is in the spotlight. The wine industry generates large amounts of waste as a consequence of grape processing operations, which can be revalued by solvent extraction of valuable antioxidants for food and fine chemical applications. Therefore, the present study focuses on the use of natural eutectic solvents (NAESs) with benign environmental, health, and safety profiles, for valorization of grape waste in the context of a circular economy. Herein, up to 15 NAESs consisting of combinations of three hydrogen bond acceptors (choline chloride, L-proline, and betaine) and four hydrogen bond donors (1,2-propanediol, glycerol, and 1,2- and 1,3-butanediol) were evaluated for antioxidant recovery. After an initial screening of the performance of NAESs by conventional extraction, the process was intensified by microwave-assisted extraction (MAE). The extracts were analyzed by UV/VIS spectrophotometric and HPLC methods. Promising results were obtained with the solvent betaine, 1,2-butanediol [1:4], using MAE at 100 °C for 3 min. Overall, the proposed NAESs-based MAE method was successfully applied to recover target compounds from grape waste, with great prospects for the antioxidants market and sustainable development for the winery sector.

## 1. Introduction

The development of new sustainable products, processes, and technologies that minimize the use of hazard chemicals is of growing interest. The use of organic solvents has negatively affected the environment and human health due to their harmful features. Although organic solvents are generally inexpensive, their high volatility and toxicity limit their applications, especially in the processing of natural products [1,2]. The huge impact of solvents in almost all industrial processes has prompted the development of greener alternatives [2,3,4]. In recent years, eutectic solvents (ESs) along with natural eutectic solvents (NAESs) formed by natural compounds are being regarded as a new generation of room temperature liquids with potential uses in various industrial fields. They are mixtures of hydrogen bond acceptors (HBAs) and hydrogen bond donors (HBDs) at different molar proportions, resulting in solvents with relatively low melting points compared with those of their individual starting components (HBAs and HBDs) [5,6,7]. In the formation of NAESs, quaternary ammonium salts, especially choline chloride and terpenes as DL-menthol, are the most common HBAs, while sugars, alcohols, carboxylic acids, or amines are used as naturally derived HBDs [7,8,9]. There are numerous possible combinations of HBAs and HBDs that can be chosen to prepare NAESs as a function of their specific application. Therefore, in most cases, it is necessary to perform an initial screening to discover the most suitable combination of HBAs and HBDs for the extraction of the compounds of interest from a specific solid biological matrix [10,11].

In general, NAESs present very attractive features such as the possibility of tailoring their physicochemical properties to a specific purpose due to their numerous structural variations, the high availability and affordability of their components, their biodegradability, their very low vapor pressure that limits air emissions, and the low toxicity of the most commonly used compounds [5,6,11,12]. As for their high viscosity that can hinder extraction processes due to mass transfer limitations, this issue can be overcome by increasing the temperature or adding solvents such as water, taking care of the stability of the mixtures [4,6,7]. Studies in the literature show that up to 30% (*v*/*v*) water addition maintains the nature of the eutectic solvent intact while significantly reducing the viscosity, generally improving the extraction process [7,13]. In addition, NAESs, presumably highly biodegradable and with low toxicity, present the advantageous possibility of being used directly in food, pharmaceutical, or cosmetic formulations [14].

The application of ESs to extract high value-added compounds from agro-industrial waste to obtain high-purity products has received increasing attention in recent years [15]. In particular, choline chloride (ChCl)-based NAESs have been demonstrated to present high affinity and stabilization of bioactive compounds, which can be correlated with the strong hydrogen bonding interactions between solutes and solvent molecules [16]. Recently, betaine has also been successfully used as HBAs in the formation of NAESs with glycerol, 1,2-propanediol, lactic acid, levulinic acid, malic acid, citric acid, glucose, and sorbitol [1]. Betaine is obtained from renewable resources, as a by-product of sugar production. Compared with commonly used ChCl, betaine is considered more attractive due to its higher biodegradability and lower toxicity profile [1,10]. Moreover, betaine-derived NAESs have been successfully used in the extraction of natural compounds from plant sources [3,17,18,19,20]. The extraction of bioactive compounds has also been tested with NAESs formed with L-proline, which is a cyclic nonessential amino acid [7,21,22,23,24]. In general, ESs and NAESs are being considered as promising solvents in separation processes of bioactive compounds from different agri-food matrices [2,4,25,26,27,28].

Bioactive compounds such as antioxidants can be extracted from different types of biomass and are subsequently widely applied in many fields [28,29,30,31]. The extraction of these has been commonly performed by solid–liquid extraction (SLE) methods with orbital or magnetic stirring [7]. However, the efficiency of classical extraction processes can be significantly improved by intensification technologies such as ultrasonic-assisted extraction (UAE) or microwave-assisted extraction (MAE) [9,15]. These extraction methodologies generally present higher efficiency, shorter extraction time, and higher purity of the extracted compounds [4]. However, there are several associated issues, including the solvents’ toxicity, thermal instability, polarity, solubility, and poor selectivity [4]. The extraction procedure of target compounds using NAESs in combination with intensification technologies is significantly affected by several factors, namely, temperature, molecular structure and composition of the ES, extraction time, water content, the use of additives, feed/solvent ratio, and pH, which plays an important role in the efficiency and yield of the process [15,32]. Furthermore, the main aspects that may influence the recovery of antioxidants using different types of natural eutectic solvents, apart from those already mentioned, would be the different chemical interactions between the structures of the compounds involved in the process, the physical properties of the solvents (such as viscosity or polarity), and the state and composition of the biomass matrix [2,4,11].

The abovementioned techniques for the separation processes can be applied to agri-food wastes with high contents of bioactive compounds, such as those derived from the wine industry. The wine sector generates large amounts of solid waste along the wine production chain, which are generally destined for cattle feed, food additives, soil conditioning, or composting, or they are trucked away to disposal sites for animal feed [33,34]. Grape pomace is a high-quality biodegradable waste product, originated during the production of must by pressing whole grapes [31,34]. The progressive increase in the amounts of wine waste represents a serious environmental pollution problem [35]. Several studies corroborate the presence of bioactive compounds remaining in this grape pomace by-product that may be of great interest for industrial applications in food, pharmaceuticals, and cosmetics [16,25,33,34]. A few ESs have been recently explored as solvents for the recovery of antioxidant compounds from grape by-products [3,5,16,36,37]. Yet the different combinations of NAESs evaluated in this study have not been systematically explored previously for this application, to our knowledge. In particular, the use of betaine or L-proline as HBAs and 1,2-propanediol or 1,2- or 1,3-butanediol as HBDs in the extraction of compounds from the evaluated matrix is pioneering. In addition, white grape waste (a mixture of skins and seeds) has been used instead of grape pomace [3], skins [5,36], or winery wastewater [37] from the processing of red variety grapes. However, the study carried out by Dabetić et al. [16] was focused on UAE, but the use of MAE technique was not evaluated, nor was a comparison between different techniques made.

As for the target compounds to be extracted, the phenolic compounds are the largest group of phytochemicals present in grape by-products. They are characterized by one or more aromatic rings bearing one or more hydroxyl groups in their chemical structure [4,7]. There are many possible classifications of phenolic compounds, but mainly, they can be divided into flavonoids (e.g., anthocyanins, flavonols, flavanols, condensed tannins, or proanthocyanidins) and non-flavonoids (e.g., phenolic acids, stilbenes, gallotannins, ellagitannins, and lignins), which are the most abundant antioxidants from natural sources [4,7]. Numerous studies suggest that these compounds work in more than one way, expressing their antioxidant activity and influencing cell communications that affect important biological processes [16]. They are well known as natural antioxidant compounds and also have potential anti-inflammatory, antiviral, analgesic, anti-carcinogenic, and antimicrobial (antifungal and antiviral) properties [7,27]. The phenolic content of wine and grape-derived waste products depends on how the grapes are processed in the winery. The phenolic composition is affected by the grape variety and degree of ripeness, as well as the agronomic and environmental conditions. Moreover, there is a significant difference between the parts of fruits (seeds, skin, and stems) considering not only the number of phenolic compounds but also their concentrations [16,38,39]. In the case of white winemaking, the grape juice ferments without the grape marc, which maintains much of its polyphenol content, predominantly flavonoids [38,40,41,42]. Therefore, herein, the possibility of recovering such antioxidants from white grape winery by-products was studied to reduce the environmental impact associated with the seasonal accumulation of this waste, through its revalorization to obtain economic and technological benefits [33,43].

In the research carried out, three natural organic compounds (choline chloride, betaine, and L-proline) were used as HBAs, and four natural organic compounds (1,2-propanediol, glycerol, and 1,2- and 1,3-butanediol) were used as HBDs to prepare different NAESs. These NAESs were evaluated as extraction agents of phenolic compounds from white grape waste (WGW) by conventional solid–liquid extraction (orbital shaker extraction (OSE)), using water and ethanol with 30% (*v*/*v*) distilled water as benchmark solvents. Such experimental screening allows a systematic evaluation of the effect of the NAESs structure on the antioxidant extraction performance (i.e., as a function of the component used as a hydrogen bond donor, the molar ratio employed for the formation of NAESs, and also, in a more unusual and novel way, in terms of the hydrogen bond acceptor component). The study continued with the intensification and optimization of the process applying microwave-assisted extraction (MAE) using the most promising solvents. The extracted phenolic compounds were quantified using UV-VIS spectrophotometric and chromatographic methods, and the antioxidant activities of the solvent extracts were also measured. The findings of this work will significantly contribute to the efficient design and selection of these novel eutectic solvents to be used in a given natural extraction process.

## 2. Results and Discussion

### 2.1. Solvents Preparation and Selection

The natural eutectic solvents (NAESs) were prepared by mixing the components according to molar ratios and conditions taken from the literature [1,32,44,45]. Table 1 shows the operating conditions (temperature and stirring time at 350 rpm) followed in the formation of the solvents that were successfully prepared. It is worth mentioning that potential NAESs composed of betaine and L-proline with 1,3-butanediol (Pro: 3But [1:4] and Bet: 3But [1:4]) could not be formed at the conditions used, as the mixture precipitated. Subsequently, only the formed solvents were used in the extraction tests. In addition, note that 30% (*v*/*v*) distilled water was added to the solvents in order to increase their polarity and decrease their viscosity. This action aimed to promote a higher affinity in the extraction of polar compounds while reducing the solubility and diffusivity limitations through the pores of the solid matrix, favoring mass transfer and the extraction process [46,47].

As for the conventional reference solvents chosen to compare the performance of NAESs, distilled water and ethanol with 30% (*v*/*v*) distilled water (Et30DW) were selected to operate under similar conditions of NAESs.

### 2.2. Total Phenolic and Flavonoid Content Extracted

**Figure 1:**
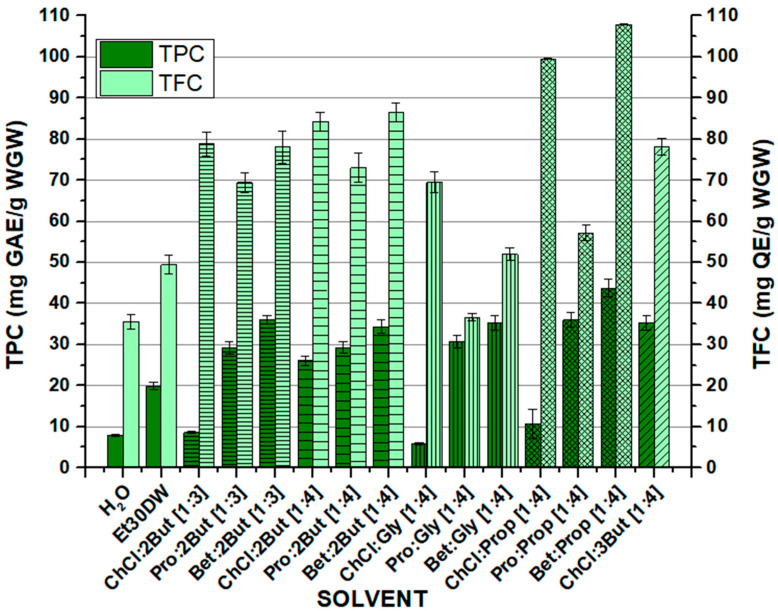
Total phenolic content (TPC, expressed as mg GAE/g WGW) and total flavonoid content (TFC, expressed as mg QE/g WGW) extracted by the solvents from white grape waste (WGW). OSE conditions: 100 min at 900 rpm, 60 °C, and 1:10 F:S ratio.

After the corresponding preparation of NAESs, a conventional solid–liquid extraction method was carried out using an orbital shaker. The process is described in detail in Section 3.2. The objective was to recover the maximum amount of phenolic compounds of interest from the white grape waste samples. Once all tests were performed with the conventional solvents (H_2_O and Et30DW) and the thirteen NAESs whose formation was possible, UV-VIS spectrophotometry methods were used to analyze the concentration of the target compounds present in the solvent extracts. Figure 1 and Table S1 (see Supplementary Material) show the results obtained from the quantification of the total phenolic content (TPC) and total flavonoid content (TFC) obtained after the extraction processes with the orbital shaker and the evaluated solvents. The quantification methods used are described in Section 3.3 and Section 3.4. The overall results indicate that the selected NAESs were able to extract the compounds of interest from the samples. In most cases, similar to or higher values than those obtained with the conventional solvents (H_2_O or Et30DW) were obtained. Furthermore, it should be noted that in all the assays, higher values were achieved for total flavonoid recovery from the samples than for total phenolic compounds (as mentioned in the introduction, the presence of flavonoids is predominant in grape-derived matrices). In addition, the Folin–Ciocalteu method is known to be significantly affected by interferences caused by other types of bioactive compounds with reducing activity that may be present in the samples, such as sugars or certain amino acids [48,49]. The TPC and TFC values of extracts obtained from the white grape waste (WGW) ranged from 5.94 ± 0.29 to 43.73 ± 2.19 mg GAE per gram of dry WGW and from 35.62 ± 1.78 to 107.88 ± 0.15 mg QE per gram of dry WGW, respectively (Figure 1). The highest total contents were extracted with Bet:Prop [1:4], while the lowest TPC was obtained with ChCl:Gly [1:4], and the lowest TFC was obtained with H_2_O.

The differences observed were presumably due to variations in the structure and physicochemical properties of the NAESs tested. The extraction capacity depended on the types of HBAs and HBDs used and the molar ratio between them. Analyzing the effect of the components on the extraction efficiency, it was observed that in the case of TPC, the results improved when using NAESs with betaine or L-proline as the HBA component, compared with using choline chloride, while for TFC, the trend was generally downward when switching from ChCl to L-proline, and a slight improvement was observed with the use of betaine. Regarding the effect of the molar ratio used, the same donor–acceptor combination was studied in the molar ratios [1:3] and [1:4], using 1-2 butanediol as donor. As shown in Figure 1, the total extracted phenolic content decreased when the ratio was [1:4], for all acceptors, except for ChCl, where it increased considerably. However, the total extracted flavonoid content was higher at a [1:4] ratio, regardless of the acceptor used. Working with a higher molar ratio seems to provide better extraction results, taking into account the total flavonoids and the error rate associated with the Folin–Ciocalteu (FC) method and considering the significant increase in total polyphenols between ClCh:1,2-butanediol [1:3] and ClCh:1,2-butanediol [1:4]. The influence of donor compound isomerism can be observed between 1,2-butanediol and 1,3-butanediol, with which, as mentioned above, it was only possible to form NAESs using choline chloride as the acceptor. In this case, the total content of extracted phenols was higher using 1,3-butanediol, while on the contrary, and with a less significant difference, the total flavonoids decreased when switching from 1,2-butanediol to 1,3-butanediol.

Moreover, it was not possible to observe a significant effect on the TPC when the results were analyzed according to the length of the donor chain. However, a trend could be observed in the case of TFC since for the same acceptor, the total flavonoid content decreased with increasing chain length from 1,2-butanediol (or 1,3-butanediol) to glycerol and increased again slightly with increasing chain length from glycerol to 1,2-propanediol.

In view of the obtained results, both for the TPC and for the TFC, it was observed that most of the studied NAESs, especially those containing betaine, provided improved results. It was shown that the evaluated solvents could be used to extract antioxidants; however, it is important to take into account that other compounds such as sugars, amino acids, or proteins could also be extracted, although probably to a lesser extent, so it would be interesting to continue with future studies in this line. Finally, it is worth mentioning that further studies are also needed to complete the analysis regarding the effect of the structure of the NAES components over the extraction of target compounds, for example, using molecular simulation to better understand their behavior.

### 2.3. Antioxidant Activity of Solvent Extracts

**Figure 2:**
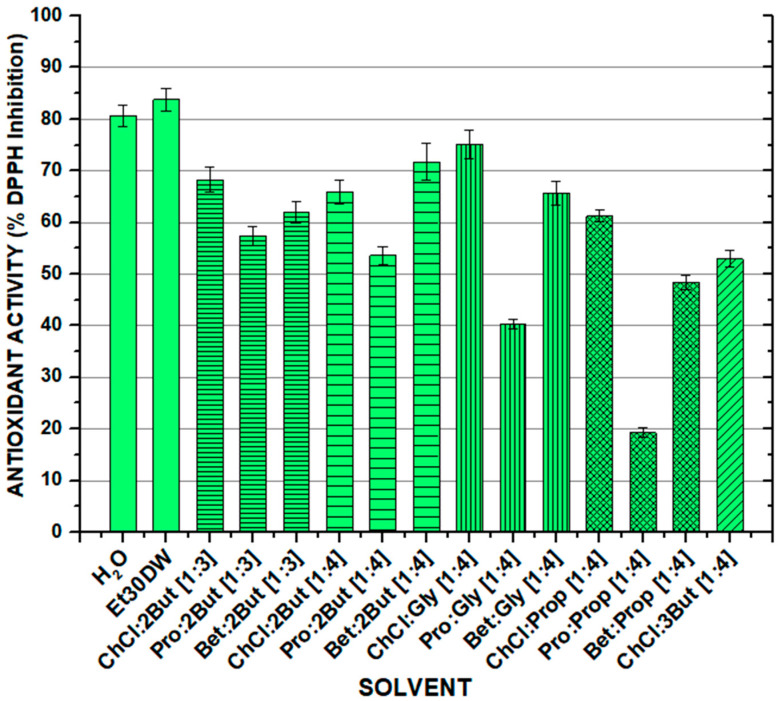
Antioxidant activity (expressed as % DPPH inhibition) of the solvent extracts obtained after the solid–liquid conventional extraction process from a white grape waste sample. OSE conditions: 100 min at 900 rpm, 60 °C, and 1:10 F:S ratio.

As for the antioxidant capacity of the extracts obtained after carrying out the orbital shaker extraction, in general, high values of antioxidant activity were obtained for all solvents, except in the cases of Pro: Gly [1:4] and Bet: Pro [1:4]. The results are shown in Figure 2 and Table S1 (see Supplementary Material). Analyzing the values in more depth, it was seen that the antioxidant capacity decreased notably when switching from choline chloride to L-proline as an acceptor compound in the formation of the NAESs, and although it increased slightly again with the use of betaine, the results did not manage to improve those of ChCl. Furthermore, the antioxidant capacity of the solvents followed the line observed in the quantification of total flavonoids (TFC), where NAESs using choline chloride as the acceptor component were the ones that offered the best results (with values achieved between 52.92 ± 1.64% (for ChCl: 3But [1:4]) and 75.09 ± 2.75% (for ChCl: Gly [1:4])). However, the values were still lower than those obtained with traditional solvents such as water Et30DW, reaching values up to 80.65 ± 2.03% and 83.81 ± 2.19%, respectively.

With regard to the effect of the molar ratio of the components forming the NAES, the antioxidant capacity decreased with a ratio of [1:4] for all acceptors except for betaine, with which it increased considerably from [1:3] to [1.4]. The influence of the isomerism of the donor compound could be seen between 1,2-butanediol (ChCl: 2But [1:4]) and 1,3-butanediol (ChCl: 3But [1:4]), where the antioxidant capacity was around 10% higher using 1,2-butanediol as the donor. Finally, it is worth mentioning that if the results were studied from the point of view of the length of the donor chain, it was not possible to observe a clear trend, as this varied considerably depending on the acceptor used.

The obtained results should be taken with caution as colorimetric methods are very susceptible to interferences in their measurement, which may overestimate or underestimate the results. Especially, precipitation was observed for NAESs formed with choline chloride, probably due to its ionic nature, which may be interfered with by the colorimetric reagents.

### 2.4. HPLC Sample Analysis

Subsequently, further analytical studies were carried out using HPLC as it is a more robust technique and is not affected by the possible interferences that can disturb the colorimetric methods.

**Figure 3:**
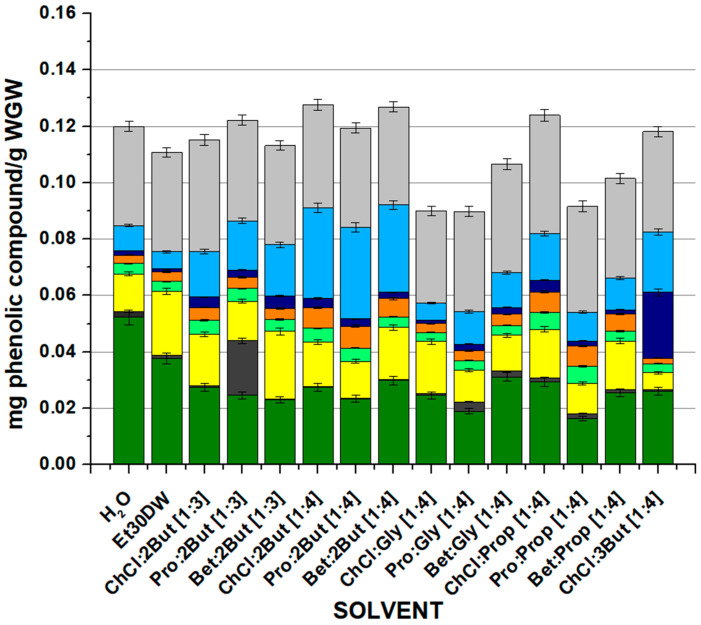
Mass (mg) of specifically each of the phenolic compounds quantified by HPLC after the conventional extraction process with each evaluated solvent. Results are expressed as mean values (mg phenolic compounds/g WGW) ± RSD (%). OSE conditions: 100 min at 900 rpm, 60 °C, and 1:10 F:S ratio.

It was sought to identify and quantify the phenolic profile of the extracts obtained by the OSE method with the evaluated solvents. Figure 3 and Table S2 (see Supplementary Material) display the extraction yields of eight relevant phenolic compounds (gallic acid, protocatechuic acid, *p*-hydroxybenzoic acid, caffeic acid, *p*-coumaric acid, ferulic acid, cinnamic acid, and quercetin) quantified by the HPLC method described in Section 3.6. The trend was very similar to that observed when measuring antioxidant capacity and flavonoids as the total content of main phenolic compounds decreased significantly when switching from chloride choline to L-proline and increased slightly when betaine was used as an acceptor. As for the quantification of the different compounds separately, very similar values were observed, with gallic acid (dark green bars) and quercetin (light grey bars) being the major compounds in all cases. Moreover, in general, the high affinity of gallic acid for water and the extraction of protocatechuic acid when Pro:2But [1:3] was used stand out. The sum of the identified compounds extracted by the solvents was in the range 0.0898 (for Pro: Gly [1:4]) to 0.1276 (for ChCl: 2But [1:4]) mg phenolic compound/g WGW for all cases. The differences in the extraction performances of the NAESs studied in this work were related to the multiple interactions between the solvents and the phenolic compounds, including hydrogen bonding interactions, polarity, and steric hindrance. The observed trends may have been due to an increased interaction of the solvents with compounds with a higher number of hydroxyl and/or aromatic groups. Generally, NAESs’ capacity to extract phenolic compounds varied considerably according to the target phenolic compounds and the solvent itself.

Overall, it was found that there were three natural eutectic solvents that offered similar results in terms of total content of major phenolic compounds extracted: ClCh, 2But [1:4] and Bet: 2But [1:4], and ClCh, Prop [1:4]. For this reason, it was decided to perform a Tukey’s test to evaluate if there were significant the variations between the three NAESs. According to the results of the test, only a small difference was observed between using ClCh: 2But [1:4] and ClCh: Prop [1:4], the former being better; accordingly, the selection was made considering only ClCh: 2But [1:4] and Bet: 2But [1:4] between which there was no significant difference in the total amount of extracted compounds. It should be noted that both eutectic solvents managed to improve the results derived from traditional extractions with water and Et30DW, recovering more of the major target phenolic compounds. However, for the following studies, the NAES Bet: 2But [1:4] (with betaine as the HBA and 1,2-butanediol as the HBD) was selected because of its good extraction performance and because betaine was considered more attractive than choline chloride due to its good ecological and economic characteristics (renewability, high biodegradability, low toxicity, and low price, among others), which favored its applicability on a larger scale [50,51].

If, as in the previous cases, the results were analyzed according to the effect of the molar ratio of the compounds forming the ES, the same acceptor–donor combination was studied in molar ratios [1:3] and [1:4]. The quantification of phenolic compounds increased with the [1:4] ratio for all acceptors except L-proline, with a very insignificant difference from [1:3] to [1:4]. As for the influence of the isomerism of the donor compound, the quantification of phenolic compounds was higher using 1,2-butanediol. As in the previous study, no clear differences were observed on the length of the donor chain.

### 2.5. Intensification of the Extraction Method

In addition to the use of environmentally friendly and safer solvents, one of the criteria for green extraction is the reduction of energy consumption by using innovative technologies such as microwave-assisted extraction. In this section, after previous confirmation of the capability of the solvents evaluated as extractive agents, the results obtained for the intensification of the extraction by using the MAE method, which is detailed in Section 3.2**,** are presented. For this purpose, the total amount of quantified phenolic compounds measured by HPLC was compared as a more robust method of analysis, both for the extraction with Et30DW and for the extraction with the selected NAES, Bet: 2But [1:4], due to its characteristics and good operational performance (up to 0.127 ± 0.005 mg phenolic compounds/g WGW could be extracted by OSE method). The operating conditions of the MAE process were varied and compared with the optimal working conditions of the OSE method (60 °C and 100 min). Two moderate operating temperatures (60 and 100 °C) and three mixing times (3, 6, and 9 min) were studied. During the assays, the temperature was controlled by continuously adjusting the microwave power output.

**Figure 4:**
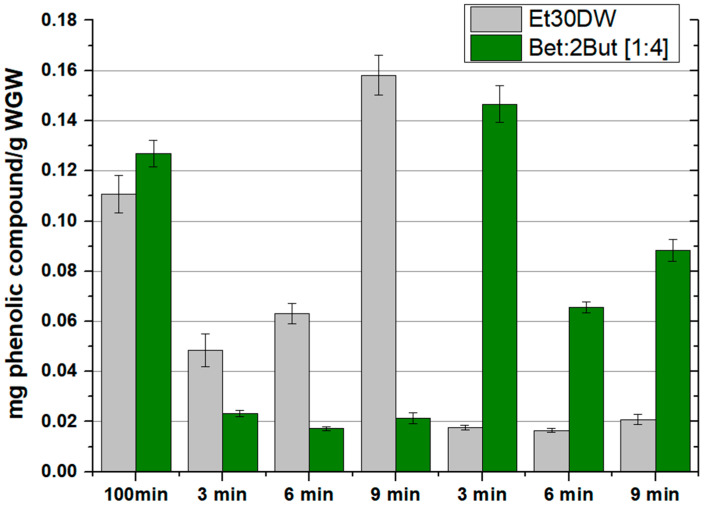
Phenolic compounds extracted according to the orbital shaker extraction (OSE) or microwave-assisted extraction (MAE) method used and as a function of the evaluated solvent (Et30DW or Bet: 2But [1:4]). Results are expressed as mean values (mg phenolic compounds/g WGW) ± RSD (%).

**Figure 5:**
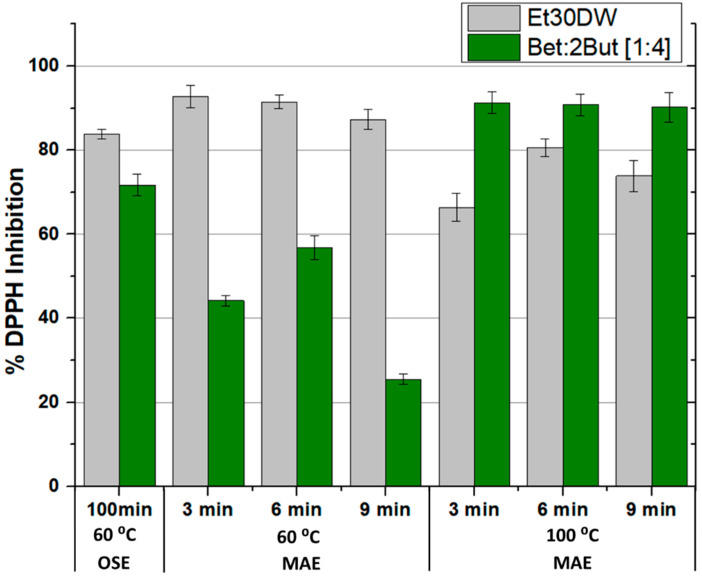
Antioxidant activity of the extracts according to the orbital shaker extraction (OSE) or microwave-assisted extraction (MAE) method used and as a function of the evaluated solvent (Et30DW or Bet: 2But [1:4]). Results are expressed as mean values (% DPPH inhibition) ± RSD (%).

The results obtained in terms of extracted phenolic compounds and antioxidant capacity of the extracts, after performing all the corresponding assays, are shown in Figure 4 and Figure 5, respectively (also in more detail in Table S3 of the Supplementary Material). A different behavior was observed between extractions with Et30DW and extractions with Bet: 2But [1:4]. With ethanol with 30% (*v*/*v*) distilled water, the total content of phenolic compounds was high when working at low temperatures (60 °C) and increased with the extraction time; however, when working at high temperatures (100 °C), the extraction efficiency decreased considerably as hardly any phenolic compounds were quantified in the extracts, regardless of the extraction time applied. Taking into account that ethanol has a boiling point of 78 °C at 1 atm and that the vial was airtight, at 100 °C, what may have happened was that the ethanol partially vaporized, forming a liquid–vapor equilibrium, and not all of it as a liquid. Therefore, if the ethanol partially vaporized, it made sense to extract less, regardless of the extraction time applied. In contrast, with the NAES, the behavior followed a completely opposite trend as at low temperature, the total content of phenolic compounds was low, with very little significant variation with increasing time. However, when the temperature was raised to 100 °C, the efficiency of NAES extractions increased at low extraction times and decreased with increasing time, which could have been due to a possible degradation of the solutes and/or solvent. This shows that if the time was short (3 min), an increase in temperature favored extraction, but care must be taken with the exposure time as the solutes and/or solvent could be degraded.

**Figure 6:**
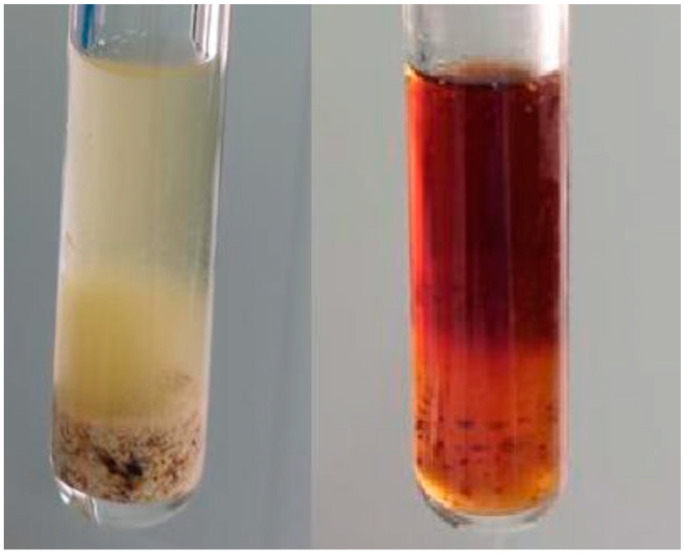
Images of the extracts obtained after undergoing the MAE process with Et30DW (**left** image) or with Bet: 2But [1:4] (**right** image). MAE conditions: 3 min at 900 rpm, 100 °C, and 1:10 F:S ratio.

The image in Figure 6 shows a visual comparison between the results obtained following the MAE process at 100 °C and 3 min, using Et30DW and Bet: 2But [1:4], where it is clear that the extract was much more concentrated in the case of NAES, which corroborated a higher extraction efficiency.

Therefore, when using MAE in combination of the eutectic solvent under study, the best results were associated with a temperature of 100 °C and a stirring time of 3 min as these conditions led to a higher extraction of total phenolic compounds. A value of 0.147 ± 0.007 mg of phenolic compound/g WGW and a DPPH inhibition percentage of 91.279 ± 2.564 was achieved. Furthermore, the use of Bet: 2But [1:4] under these optimal conditions significantly improved the results obtained in MAE in comparison with Et30DW (under similar MAE conditions), as well as the results obtained in conventional orbital shaker extraction (OSE), either with NAES itself or with the traditional solvent. Moreover, the energy required when using the microwave reactor for 3 min with a maximum operating power of 850 W compared with the orbital shaker for 100 min with an operating power of 515 W, 153 kJ versus 3090 kJ, was up to 20 times less. Thus, the developed NAES-based MAE method improved the extraction performance of the evaluated solvents to recover high-value antioxidants from grape-derived waste, while the intensification of the extraction process saved time, costs, and energy.

## 3. Materials and Methods

### 3.1. Chemicals and Materials

The plant material matrix used was a white grape waste formed by the skins and seed of the table grape variety *Doña María*, a type of white grape native to Spanish territory. The grape residue was ground and dried at 50 °C in an oven for 24 h before use.

**Figure 7:**
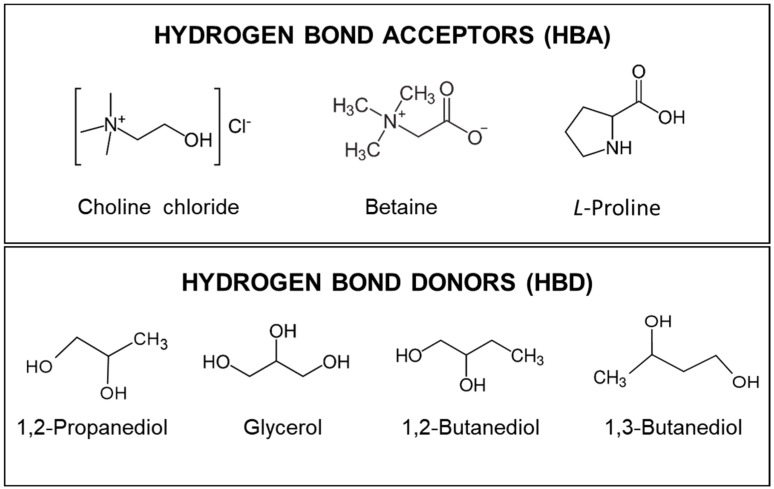
Chemical structures of the components of NAESs (HBAs and HBDs).

For the preparation of the natural eutectic solvents, the following compounds were used as HBAs: choline chloride (C_5_H_14_ClNO, 99 wt%, CAS No. 67-48-1), L-proline (C_5_H_9_NO_2_, 99 wt%, CAS No. 147-85-3), and betaine (C_5_H_11_NO_2_, 98 wt%, CAS No. 107-43-7). In parallel, the following compounds were used as HBDs: 1,2-propanediol (C_3_H_8_O_2_, 99.5 wt%, CAS No. 57-55-6), glycerol (C_3_H_8_O_3_, 99 wt%, CAS No. 56-81-5), 1,2-butanediol (C_4_H_10_O_2_, 98 wt%, CAS No. 584-03-2), and 1,3-butanediol (C_4_H_10_O_2_, 99 wt%, CAS No. 107-88-0). Figure 7 shows the chemical structure of the compounds used for the prepared NAESs. All NAESs and ethanol (C_2_H_6_O, 99 wt%, CAS No. 64-17-5) were used with 30% (*v*/*v*) water.

Moreover, gallic acid (C_7_H_6_O_5_, 97.5 wt%, CAS No. 149-91-7), protocatechuic acid (C_7_H_6_O_4_, 97 wt%, CAS No. 99-50-3), *p*-hydroxybenzoic acid (C_7_H_6_O_3,_ 99 wt%, CAS No. 99-96-7), caffeic acid (C_9_H_8_O_4_, 98 wt%, CAS No. 331-39-5), *p*-coumaric acid (C_9_H_8_O_3_, 98 wt%, CAS No. 501-98-4), ferulic acid (C_10_H_10_O_4_, 99 wt%, CAS No. 537-98-4), cinnamic acid (C_9_H_8_O_2_, 99 wt%, CAS No. 140-10-3), and quercetin (C_15_H_10_O_7_, 95 wt%, CAS No. 117-39-5) were mainly used in the preparation of standard solutions for the specific quantification of the phenolic compounds present in the samples.

Finally, a number of reagents were necessary to carry out the analytical methods: aluminum chloride (AlCl_3_, 98 wt%, CAS No. 7446-70-0), sodium hydroxide (NaOH, 99 wt%, CAS No. 1310-73-2), sodium carbonate (Na_2_CO_3_, 99.5 wt%, CAS No. 497-19-8), sodium nitrate (Na_2_NO_3_, 99.5 wt%, CAS No. 7631-99-4), 2,2-diphenyl-1-picrylhydrazyl or DPPH (98 wt%, CAS No. 1898-66-4), Folin–Ciocalteu reagent (F9252), acetic acid glacial (99 wt%, CAS No. 64-19-7), water (HPLC grade, CAS No. 7732-18-5), and acetonitrile (HPLC grade, CAS No. 75-05-8).

All used solvents and reagents were provided by Sigma Aldrich (Madrid, Spain).

### 3.2. Extraction Methods

Extractions were initially carried out following a conventional solid–liquid extraction method. The dried white grape waste samples were placed in contact with the solvents in an orbital shaker incubator VorTemp 1550 (Labnet, Madrid, Spain) under the following conditions: 100 min of shaking at 900 rpm, 60 °C, and a feed: solvent ratio of 1:10 (0.5 g dried grape residue, 5 mL solvent). It was decided to apply an extraction temperature and time that would increase the solubility and diffusion coefficients of the phenolic compounds present in the grape, trying to reduce as much as possible the surface tension and viscosity of the solvents to favor the mass transfer. The selected values were based on preliminary studies carried out by our research group. Working with high extraction temperatures can lead to degradation of the phenolic compounds to be extracted due to the occurrence of redox reactions, polymerizations, or hydrolysis, so the limit was set at 60 °C for all extractions [48,52]. Therefore, a higher temperature was not tested, also due to the long operating time used.

Subsequently, the process was intensified by performing a microwave-assisted extraction instead of the conventional extraction method. For the tests performed, the white grape waste and the volume of solvent selected as the best extraction agent were mixed in a glass vial and placed into the Monowave 400 microwave reactor (Anton Paar, Madrid, Spain). The extraction temperature and time were then adjusted. The evaluated operating conditions were the applied temperature (60 °C and 100 °C) and the extraction time (3, 6, and 9 min). The aim was to evaluate which power (temperature-related) and which time provided the best extraction results and whether or not they improved the results derived from the OSE method.

**Figure 8:**
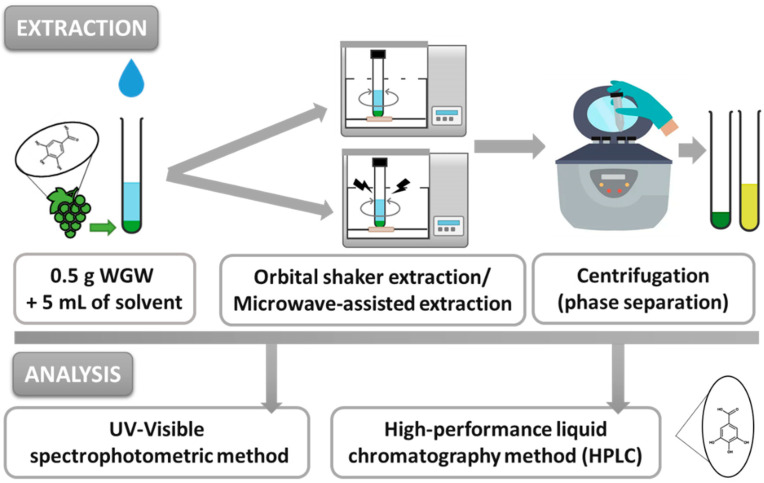
Global procedure: methods of extraction and analysis of phenolic compounds from white grape waste (WGW) by evaluating conventional solvents and NAESs as extraction agents.

After all the extraction assays, the samples were centrifuged at 3500 rpm for 15 min in a Unicen 21 Centrifuge (Ortoalresa, Madrid, Spain) to separate the phases. The liquid extracts were then analyzed by UV-VIS spectrophotometry and high-performance liquid chromatography (HPLC) analytical methods to quantify the compounds of interest. The procedures were also performed using Et30DW as a conventional organic solvent, for comparative purposes. The assays were performed in triplicate, and the mean values were calculated and are shown in this work. Figure 8 shows a schematic representation of the main steps involved in the evaluated extraction procedures.

### 3.3. Total Phenolic Content (TPC)

To quantify the total phenolic content (TPC) present in each extract, the Folin–Ciocalteu (FC) method was used [37,53]. This method is mainly based on the redox reaction at basic pH of the total phenolic compounds present in the extract with the Folin–Ciocalteu reagent, which, after being reduced by the phenolic groups present, turns from a yellow to a blue color, the intensity of which depends on the total amount of phenolic compounds.

Briefly, 100 μL of sample and 100 μL of Folin–Ciocalteu reagent were mixed. This mixture was homogenized and incubated in the dark and at room temperature for 3 min, after which 2 mL of the 2% by weight Na_2_CO_3_ solution was added, and the mixture was left to stand for half an hour in the dark. After this time, the measurements were carried out at a wavelength of 765 nm with the UV-VIS spectrophotometer V-730 (Jasco, Spain), giving the absorbance data.

Subsequently, the concentrations of total phenolic compounds in each sample were calculated using the calibration line (y = 0.0045x + 0.0317 R^2^ = 0.9946) previously obtained with gallic acid standard solutions at known concentrations (0–160 mg/L). The TPC results were expressed as mg gallic acid equivalents (GAE) per gram of dry white grape waste (mg GAE/g WGW) ± relative standard deviation (RSD, %). Measurements were carried out in triplicate, and the dilution factor was taken into account in case it was necessary to dilute the samples for their quantification. Moreover, the possible minimum or null absorbance of the solvents alone was measured, and the values were considered.

### 3.4. Total Flavonoid Content (TFC)

The estimation of total flavonoids present in the grape extract was carried out following the method described by Zhishen, Mengcheng, and Jianming (1999) [54] and further modified by Jahanban-Esfahlan and Jamei (2012) [55] and Yildiz et al. (2014) [56]. This method consisted of reacting the flavonoid molecules present in a given sample with aluminum ions, resulting in a red reaction product whose color intensity depended on the number of hydroxyl groups present in the flavonoid molecules of the sample and the properties of the aluminum ions.

The reagents were added to a 10 mL volumetric flask in the following order: 2 mL of distilled water were added to each of the test tubes, followed by 150 μL of Na_2_NO_3_ and 450 μL of sample. The mixture was thoroughly shaken and incubated for five minutes at room temperature, followed by the addition of 150 μL of AlCl_3_, shaking the tubes and leaving them to stand for a further five minutes at room temperature. Finally, 1 mL of 1 M NaOH was added to each tube to terminate the reaction. The reaction solution was mixed manually, and it was kept at room temperature for 15 min. The absorbance of the reaction mixture was subsequently measured at 510 nm by using the UV-VIS spectrophotometer V-730.

Finally, the total flavonoid content in each extract sample was calculated using the calibration line (y = 0.0008x + 0.0014 R^2^ = 0.9979) previously obtained with quercetin standard solutions at known concentrations (0–120 mg/L). The TFC results were expressed in milligrams of quercetin equivalents (QE) per gram of dry white grape waste (mg QE/g WGW) ± RSD (%). Measurements were carried out in triplicate, and the dilution factor was taken into account in case it was necessary to dilute the samples for their quantification. Moreover, the possible minimum or null absorbance of the solvents alone was measured, and the values were considered.

### 3.5. Antioxidant Activity

The antioxidant activity of phenolic compounds present in the obtained NAES extracts was evaluated using the DPPH analysis method described by Brand Williams et al. [57]. It consisted of bringing the extract to be analyzed into contact with the free radical present in the DPPH; the extract acted as a free radical scavenger, giving rise to a reaction that turned the blue-violet color of the DPPH toward yellow depending on the antioxidant capacity of the extract. In this way, and taking the antioxidant capacity of a methanol sample as a reference, it was possible to know the real antioxidant capacity of the sample to be analyzed by the difference in absorption.

The samples (100 μL) were mixed and allowed to react with 2.9 mL of the DPPH radical solution (6 × 10^−5^ mol/L). Then, the mixtures were shaken, and after incubation in the dark (30 min at room temperature), the absorbance at 515 nm was measured with the UV-VIS spectrophotometer V-730.

Finally, the percentage inhibition of each extract, i.e., the antioxidant capacity, was calculated, taking into account that the real absorbance of each extract was the difference between the measured value and the absorbance value of the methanol sample as a blank.

### 3.6. HPLC Phenolic Content Analysis

The concentrations of compounds of interest extracted by the NAES were determined using an Agilent 4000 series HPLC system (JASCO, Madrid, Spain) equipped with a quaternary pump, a DAD detector, an auto sampler, and a thermostatted column compartment. A Fortis C18 column (250 mm × 4.6 mm, 5 μm) was used to detect chromatographic separations at room temperature (289.15 K). The mobile phase used was water/formic acid (98.75:1.25, *v*/*v*) (phase A) and acetonitrile (phase B). Gradient conditions were as follows: 10–20% B linear 0–8 min, 20–50% B linear 8–15 min, 50–70% B linear 15–20 min, 70–10% B linear from 20 to 25 min with re-equilibration of the column for 5 min under initial gradient conditions, and flow rate of 0.8 mL/min. Another 10 min post-run time was carried out to fully equilibrate the system. The sample injection volume was 20 µL. UV-VIS spectra were measured in the wavelength range of 200–600 nm.

The HPLC methodology developed was used to quantify the target phenolic compounds present in the extracts obtained from the white grape sample under study: gallic acid, protocatechuic acid, *p*-hydroxybenzoic acid, caffeic acid, *p*-coumaric acid, ferulic acid, cinnamic acid, and quercetin. The identification and peak assignment of these compounds were based on the comparison of their retention times and spectral data with those of the authentic standards. Quantification was carried out by the external standard method at the wavelength of maximum absorbance for each compound as listed below (Table 2). The table shows the retention times, wavelengths, and calibration lines of each of the compounds to be identified and quantified. Three replicates from each sample were analyzed, and the results were expressed as mg compound/L, which was subsequently converted into mg compound/g WGW.

## 4. Conclusions

The present study evaluated the application of novel natural eutectic solvents (NAESs) in the revaluation of white grape waste by recovering natural phenolic antioxidants. A systematic assessment of the effect of both the donor and acceptor components as well as the ratio used to form the NAESs was performed. The solid–liquid extraction of antioxidants from white grape waste using NAESs in an orbital shaker (60 °C and 100 min) offered better results than the use of traditional volatile organic solvents such as Et30DW (ethanol with 30% (*v*/*v*) distilled water). In particular, the best results were obtained with Bet: 2But [1:4] at 0.127 ± 0.005 mg of phenolic compounds/g white grape waste, gallic acid and quercetin being the main antioxidants present in the extracts. Afterward, the performance of the selected NAES was further improved by process intensification using microwave-assisted extraction (MAE). In fact, with MAE technology, an enhanced antioxidant extraction was achieved in a much shorter time (100 °C and 3 min), with an energy consumption up to 20 times lower. Thus, the NAESs-based MAE method developed herein managed to extract target high-value bioactive compounds increasing the sustainability and profitability of the winery sector, while developing an intensified processes based on alternative solvents in the context of green chemistry to boost the circular bioeconomy.

## Figures and Tables

**Table 1:** Conditions and observations of NAES formation.

Nomenclature of NAES	HBA	HBD	Molar Relation	Temp. (°C)	Time (h)
ChCl: 2But [1:3]	Choline chloride	1,2-butanediol	1:3	100	2
Pro: 2But [1:3]	L-Proline	1,2-butanediol	1:3	100	2
Bet: 2But [1:3]	Betaine	1,2-butanediol	1:3	100	2
ChCl: 2But [1:4]	Choline chloride	1,2-butanediol	1:4	60	6
Pro: 2But [1:4]	L-Proline	1,2-butanediol	1:4	80	2
Bet: 2But [1:4]	Betaine	1,2-butanediol	1:4	70	6
ChCl: Gly [1:4]	Choline chloride	Glycerol	1:4	60	4
Pro: Gly [1:4]	L-Proline	Glycerol	1:4	60	4
Bet: Gly [1:4]	Betaine	Glycerol	1:4	60	6
ChCl: Prop [1:4]	Choline chloride	1,2-propanediol	1:4	60	2
Pro: Prop [1:4]	*L*-Proline	1,2-propanediol	1:4	60	3
Bet: Prop [1:4]	Betaine	1,2-propanediol	1:4	60	4
ChCl: 3But [1:4]	Choline chloride	1,3-butanediol	1:4	60	6

**Table 2:** Parameters of HPLC analysis of eight target phenolic compounds in an aqueous standard sample (measurement wavelength, retention time, and parameter values of the calibration lines for the compounds at concentrations between 0 and 6 mg/L).

Phenolic Compound	Wavelength (nm)	Retention Time (min)	Calibration Line x: [0.1–5.2 mg/L]	R^2^
Gallic acid	271	7.91	y = 68324x − 3976.4	0.9984
Protocatechuic acid	257	12.46	y = 84529x	0.9992
*p*-Hydroxybenzoic acid	257	16.42	y = 179176x − 6177.7	0.9993
Caffeic acid	323	16.63	y = 139266x − 4224.5	0.9987
*p*-Coumaric acid	323	18.63	y = 145402x − 2681.4	0.9993
Ferulic acid	323	18.90	y = 142415x − 864.88	0.9991
Cinnamic acid	271	22.50	y = 61849x − 18147	0.9873
Quercetin	370	21.43	y = 194840x	0.9997

## Data Availability

The data in this study are available in the article.

## Supplementary Materials

The following supporting information can be downloaded at https://www.mdpi.com/article/10.3390/molecules28031153/s1. Table S1: Total phenolic content, total flavonoid content and antioxidant activity of the solvent extracts. Table S2: Mass of each phenolic compounds quantified by HPLC after the conventional extraction process with each solvent evaluated. Table S3: Phenolic compounds and antioxidant activity of the extracts according to the orbital shaker extraction or micro-wave-assisted extraction method.

## Sample Availability

Samples of the compounds are no longer available from the authors.
