# Supplementary material for: Enhanced Recovery of Natural Antioxidants from Grape Waste Using Natural Eutectic Solvents-Based Microwave-Assisted Extraction
Source: Molecules. 2023 Jan 24;28(3):1153. doi: 10.3390/molecules28031153 (PMC9920075; doi:10.3390/molecules28031153)
Supplement: Supplementary file 1 [file molecules-28-01153-s001.zip › molecules-2154274-supplementary.pdf]

SUPPLEMENTARY MATERIAL

Enhanced Recovery of Natural Antioxidants from Grape Waste Using Natural Eutectic Solvents-Based Microwave-Assisted Extraction

Raquel Cañadas, Blanca Sáenz de Miera, Paloma Méndez, Emilio J. González and María González-Miquel *

Department of Chemical and Environmental Engineering, Higher Technical School of Industrial Engineering, Universidad Politécnica de Madrid. C/José Gutiérrez Abascal 2, 28006 Madrid, Spain
* Correspondence: maria.gonzalezmiquel@upm.es

Supplementary data includes: 3 Tables

Table S1. Total phenolic content (TPC, mg GAE/g WGW) and total flavonoid content (TFC, mg Q/g WGW) extracted by the solvents evaluated from a white grape waste sample (WGW) and antioxidant activity (% DPPH inhibition) of the solvent extracts. Results are expressed as mean values ± relative standard deviation (RSD, %). OSE conditions: 100 min at 900 rpm, 60 °C and 1:10 F:S ratio.

SOLVENTS	TPC (mg GAE/g WGW) ± RSD (%)	TFC (mg Q/g WGW) ± RSD (%)	Antioxidant activity (% DPPH inhibition) ± RSD (%)
H_2O	7.89 ± 0.19	35.62 ± 1.78	80.65 ± 2.03
Et30DW	19.92 ± 0.96	49.40 ± 2.27	83.81 ± 2.19
ChCl: 2But [1:3]	8.643 ± 0.14	78.81 ± 2.94	68.28 ± 2.41
Pro: 2But [1:3]	29.26 ±1.46	69.39 ± 2.47	57.38 ± 1.87
Bet: 2But [1:3]	36.02 ± 1.10	78.07 ± 3.90	62.00 ± 2.10
ChCl: 2But [1:4]	26.06 ±1.20	84.27 ± 2.21	65.85 ± 2.29
Pro: 2But [1:4]	29.31 ±1.47	73.04 ± 3.65	53.58 ± 1.68
Bet: 2But [1:4]	34.36 ± 1.72	86.46 ± 2.32	71.71 ± 3.58
ChCl: Gly [1:4]	5.94 ± 0.29	69.51 ± 2.48	75.09 ± 2.75
Pro: Gly [1:4]	30.78 ± 1.54	36.65 ± 0.83	40.30 ± 1.01
Bet: Gly [1:4]	35.32 ± 1.77	51.97 ± 1.60	65.62 ± 2.28
ChCl: Prop [1:4]	10.76 ± 3.54	99.50 ± 0.12	61.26 ± 1.06
Pro: Prop [1:4]	35.99 ± 1.79	57.17 ± 1.86	19.26 ± 0.96
Bet: Prop [1:4]	43.73 ± 2.19	107.88 ± 0.15	48.33 ± 1.42
ChCl: 3But [1:4]	35.25 ± 1.76	78.13 ± 1.91	52.92 ± 1.64

Table S2. Mass of each phenolic compounds quantified by HPLC after the conventional extraction process with each solvent evaluated. Results are expressed as mean values (mg phenolic compounds/g WGW) ± RSD (%). Phenolic compounds: gallic acid (GA), protocatechuic acid (PA), *p*-hydroxybenzoic acid (HA), caffeic acid (CA), *p*-coumaric acid (*p*CA), ferulic acid (FA), cinnamic acid (CiA) and quercetin (Q). OSE conditions: 100 min at 900 rpm, 60 °C and 1:10 F:S ratio.

SOLVENTS	Mass (mg) of phenolic compound recovered / g WGW ± RSD (%)							
	GA	PA	HA	CA	*p*CA	FA	CiA	Q
H_2O	0.0522 ± 0.0026	0.0019 ± 0.0001	0.0135 ± 0.0007	0.0037 ± 0.0002	0.0029 ± 0.0001	0.0016 ± 0.0001	0.0090 ± 0.0005	0.0351 ± 0.0018
Et30DW	0.0377 ± 0.0019	0.0011 ± 0.0001	0.0228 ± 0.0010	0.0034 ± 0.0002	0.0035 ± 0.0002	0.0010 ± 0.0000	0.0061 ± 0.0003	0.0353 ± 0.0018
ChCl: 2But [1:3]	0.0275 ± 0.0014	0.0004 ± 0.0000	0.0183 ± 0.0007	0.0049 ± 0.0002	0.0046 ± 0.0002	0.0036 ± 0.0002	0.0161 ± 0.0008	0.0397 ± 0.0020
Pro: 2But [1:3]	0.0246 ± 0.0012	0.0194 ± 0.0001	0.0140 ± 0.0012	0.0045 ± 0.0002	0.0039 ± 0.0002	0.0026 ± 0.0001	0.0175 ± 0.0009	0.0356 ± 0.0018
Bet: 2But [1:3]	0.0230 ± 0.0012	0.0002 ± 0.0000	0.0241 ±	0.0042 ± 0.0002	0.0039 ± 0.0002	0.0044 ± 0.0002	0.0182 ± 0.0009	0.0352 ± 0.0018
ChCl: 2But [1:4]	0.0273 ± 0.0014	0.0004 ± 0.0000	0.0157 ± 0.0008	0.0048 ± 0.0002	0.0073 ± 0.0002	0.0034 ±0.0002	0.0321 ± 0.0016	0.0365 ± 0.0018
Pro: 2But [1:4]	0.0234 ± 0.0012	0.0003 ± 0.0000	0.0130 ± 0.0006	0.0048 ± 0.0002	0.0077 ± 0.0004	0.0026 ± 0.0001	0.0325 ± 0.0016	0.0352 ± 0.0018
Bet: 2But [1:4]	0.0298 ± 0.0015	0.0004 ± 0.0000	0.0184 ± 0.0009	0.0039 ± 0.0002	0.0064 ± 0.0004	0.0022 ± 0.0001	0.0310 ± 0.0003	0.0348 ± 0.0017
ChCl: Gly [1:4]	0.0246 ± 0.0012	0.0007 ± 0.0000	0.0184 ± 0.0009	0.0033 ± 0.0002	0.0031 ± 0.0003	0.0012 ± 0.0001	0.0060 ± 0.0006	0.0329 ± 0.0016
Pro: Gly [1:4]	0.0189 ± 0.0009	0.0033 ± 0.0002	0.0112 ± 0.0006	0.0034 ± 0.0002	0.0036 ± 0.0002	0.0023 ± 0.0001	0.0115 ± 0.0006	0.0356 ± 0.0018
Bet: Gly [1:4]	0.0310 ± 0.0016	0.0020 ± 0.0001	0.0128 ± 0.0006	0.0035 ± 0.0002	0.0039 ± 0.0002	0.0025 ± 0.0001	0.0124 ± 0.0008	0.0385 ± 0.0019
ChCl: Prop [1:4]	0.0293 ± 0.0015	0.0016 ± 0.0001	0.0172 ± 0.0009	0.0059 ± 0.0003	0.0074 ± 0.0002	0.0041 ± 0.0002	0.0164 ± 0.0005	0.0421 ± 0.0021

**Pro: Prop [1:4]**	0.0165 ± 0.0008	0.0016 ± 0.0001	0.0107 ± 0.0005	0.0060 ± 0.0003	0.0073 ± 0.0004	0.0017 ± 0.0001	0.0103 ± 0.0001	0.0375 ± 0.0019
**Bet: Prop [1:4]**	0.0255 ± 0.0013	0.0012 ± 0.0001	0.0172 ± 0.0009	0.0035 ± 0.0002	0.0060 ± 0.0003	0.0016 ± 0.0001	0.0112 ±0.0006	0.0353 ± 0.0018
**ChCl: 3But [1:4]**	0.0260 ± 0.0013	0.0005 ± 0.0000	0.0060 ± 0.0003	0.0033 ± 0.0002	0.0019 ± 0.0001	0.0234 ± 0.0002	0.0215 ± 0.0011	0.0355 ± 0.0018

Table S3. Phenolic compounds extracted (mg phenolic compounds/g WGW) and antioxidant activity (% DPPH Inhibition) of the extracts according to the orbital shaker extraction (OSE) or micro-wave-assisted extraction (MAE) method used and as a function of the solvent evaluated (ethanol with 30 % (v/v) distilled water (Et30DW) or Bet: 2But [1:4]). Results are expressed as mean values ± RSD.

SOLVENTS	EXTRACTION METHOD	TEMPERATURE (ºC)	TIME (min)	Mass (mg) of phenolic compounds recovered / g WGW ± RSD (%)	Antioxidant activity (% DPPH inhibition) ± RSD (%)
**Et30DW**	OSE	60	100	0.111 ± 0.008	83.811 ± 1.191
	MAE	60	3	0.048 ± 0.006	92.834 ± 2.642
			6	0.063 ± 0.004	91.508 ± 1.575
			9	0.158 ± 0.008	87.303 ± 2.365
		100	3	0.018 ± 0.008	66.390 ± 3.319
			6	0.017 ± 0.008	80.604 ± 2.030
			9	0.021 ± 0.002	73.834 ± 3.692
**Bet: 2But [1:4]**	OSE	60	100	0.127 ± 0.005	71.710 ± 2.585
	MAE	60	3	0.023 ± 0.002	44.180 ± 1.209
			6	0.017 ± 0.001	56.828 ± 2.841

			9	0.021 ± 0.002	25.505 ± 1.275
		100	3	0.147 ± 0.007	91.279 ± 2.564
			6	0.066 ± 0.002	90.785 ± 2.539
			9	0.088 ± 0.004	90.245 ± 3.512
